# GA-AFedOD: gradient-aligned active federated learning for resource-aware object detection in edge industrial IoT

**DOI:** 10.3389/frai.2026.1895239

**Published:** 2026-08-10

**Authors:** Zepeng Wang, Xiaogang Yuan, Jie Chen

**Affiliations:** 1 School of Cyberspace Security, Gansu University of Political Science and Law, Lanzhou, China; 2 College of Electronic Engineering, National University of Defense Technology, Hefei, China

**Keywords:** active learning, edge intelligence, federated learning, industrial IoT, object detection

## Abstract

Visual object detection is essential for defect inspection and process monitoring in edge-deployed Industrial Internet of Things (IIoT). Yet, training accurate detectors across distributed factories faces stringent constraints on data privacy, annotation budgets, and uplink communication. Standard federated learning (FL) preserves locality but often wastes labeling resources on redundant frames and overlooks detection-specific gradient alignment when scheduling clients. To bridge this gap, we propose Gradient-Aligned Active Federated Object Detection (GA-AFedOD), a unified framework that jointly optimizes annotation selection, client participation, and model aggregation as a constrained stochastic program. A novel utility metric integrates box-level uncertainty, prototype diversity, gradient alignment, and resource pricing, enabling edge clients to perform locally guided active querying while the server solves a lightweight primal-dual problem for budget-aware client scheduling. We prove a submodular approximation guarantee for the greedy sampling rule and establish a non-convex convergence bound that explicitly captures the impact of label budgets, client drift, and compression noise. This article further clarifies the relationship with recent federated active learning and industrial detection studies, adds parameter and theory-diagnostic analyses, and distinguishes controlled simulation evidence from real-world deployment validation on industrial datasets such as RasPiDets, Electric Power Fitting Dataset (EPFD), and Diverse Insulator Dataset (DINS). Controlled simulation results show that GA-AFedOD achieves considerably higher mean average precision (mAP) while reducing both annotation costs and uplink consumption by over 40% compared with competitive baselines.

## 1 Introduction

Industrial visual intelligence is moving from cloud-centric analytics to edge-deployed perception (Reis, 2025). Cameras, sensors, and embedded accelerators now inspect products, guide robots, monitor hazardous zones, and identify anomalies near production lines (Xie et al., 2024; Liu X. et al., 2025). This shift brings several distinct challenges. Data come from many factories and machines, with visual domains varying by illumination, lens position, product batches, and defect rarity (Yao et al., 2024; Wang et al., 2024). Labeling is expensive because object detection needs bounding boxes, not just image-level tags (Zhao C. et al., 2024). Edge devices also have limited memory, energy, and bandwidth. These factors make efficient object detection for Industrial Internet of Things (IIoT) privacy-sensitive, resource-constrained, and continuously changing.

Federated learning (FL) offers a natural starting point: clients keep raw data local and share only model updates (Cui et al., 2025). Early FL work introduced FedAvg and studied the impact of non-IID client data on distributed optimization (Alotaibi et al., 2024). Later methods reduced client drift using control variates, proximal regularization, batch-normalization localization, and dynamic regularizers (Zhang H. et al., 2025; Robalino-Díaz et al., 2026). Industrial FL research has linked these ideas to IIoT, digital twins, and 5G edge networks (Tao et al., 2026). Recent work also explores active client selection, stale updates, robust aggregation, split learning, and decentralized sporadic participation (Bouaggad and Grabar, 2025; Qiao et al., 2025).

However, standard FL remains insufficient for edge object detection. First, detection losses are multi-component objectives; gradients for classification, localization, and objectness can conflict. A client beneficial for classification may not aid rare-defect localization (Liu D. et al., 2025). Second, active learning for detection is inherently harder than for classification, as uncertainty must be evaluated across images, boxes, and classes (Zhao et al., 2025; Khan et al., 2021). Third, locally selected active sets may lack global utility under non-IID factory conditions (Jiang et al., 2021; Xu et al., 2024). Recent federated active learning and object-detection studies have made progress, yet many decouple sample querying from client scheduling and ignore edge resource prices, update alignment, and detector-specific localization uncertainty (Li et al., 2024; Ma et al., 2025).

Recent research highlights those gaps from various angles. Inconsistency-based federated active learning improves weakly supervised selection under heterogeneity, and client heterogeneity-aware or foundation-model-assisted methods reduce annotation and communication burdens (Zong et al., 2025; Zhang J. et al., 2025; Li H. et al., 2025). Personalized and robust FL methods improve aggregation amid conflicts, stale updates, or adversarial noise (Wang et al., 2025). For detection, efficient detectors, training-free long-tail detection, diffusion-generated data, and test-time adaptive pruning push accuracy-efficiency tradeoffs forward (Mo et al., 2026). Asynchronous split FL, decentralized sporadic FL, and 5G edge FL further demonstrate the need to tolerate heterogeneous computation and communication (Ao et al., 2025). IIoT reviews and clustered FL research emphasize that edge FL must control latency, energy, and non-IID industrial data rather than optimizing accuracy alone (Fan et al., 2024). Deployment-oriented federated detection and active detection toolchains underscore the need for detector-specific evaluation (Liu et al., 2020; Deng et al., 2022; Chai et al., 2024). Those works motivate a unified method treating labels, clients, and communication as coupled variables rather than isolated modules.

We also incorporated recent industrial detection and uncertainty-modeling studies suggested by the reviewers. RasPiDets, Electric Power Fitting Dataset (EPFD), and (DINS) highlight that real inspection data include strong cross-device variations, small objects, long-tailed categories, and end-edge-cloud latency constraints (Liang et al., 2025; Yang et al., 2025; Cui et al., 2024). Active uncertainty estimation and multi-view/multimodal uncertainty modeling have been investigated in imbalanced active learning, evidential multimodal recognition, pseudo-label graph learning, graph-consistent multi-view fusion, and tensor-based semi-supervised multi-view clustering (Chen W. et al., 2026; Fu et al., 2026; Li et al., 2026; Li G. et al., 2025; Yu et al., 2026). Meanwhile, industrial detection models such as YOLOv8 Deep Transfer Learning (YOLOv8DTL), pantograph slide plate monitoring (PSPMoni), and pre-trained spatial-temporal (PST)-Transformer show that task-specific feature transfer and temporal/spatial modeling are important in few-shot rail abrasion detection, pantograph wear monitoring, and driving-pose estimation (Zhang Z. et al., 2025; Yao et al., 2025; Zhao H. et al., 2024). These works strengthen the motivation for an active federated detector whose sample utility is not merely uncertainty-based but also tied to global descent alignment, localization uncertainty, and resource prices.

We also position Gradient-Aligned Active Federated Object Detection (GA-AFedOD) within the broader distributed-optimization and game-theoretic resource-allocation literature. Recent studies formulate sensor coverage through a potential game, use policy optimization for power-efficient multi-agent task allocation, and develop distributed game mechanisms for graph-covering problems (Chen et al., 2026a,b, 2025a,b,c). The Hydro-Intelligence Body further illustrates how multi-modal sensing architectures can coordinate heterogeneous sensing and utility signals in a resource-aware autonomous system (Chen M. et al., 2026). These works are complementary rather than direct federated-active-learning baselines: they motivate locally informed distributed decisions, whereas our objective jointly prices label acquisition, client participation, and detector update quality under a time-varying federated budget.

The core advantage of the proposed method in this article is embedding active learning directly into federated optimization through gradient alignment. Rather than selecting samples solely by entropy or clients solely by data size, GA-AFedOD estimates how a queried image shifts the expected descent direction of the global detector. It prioritizes unlabeled frames that are simultaneously uncertain, feature-diverse, and aligned with the server-side detection gradient, while penalizing costly clients and saturated domains. This design is particularly relevant for IIoT, where rare defects often occur in isolated factories and communication bottlenecks are device-specific and time-varying.

This study develops a complete framework for active federated object detection in edge-deployed IIoT. We define a constrained federated detection objective with annotation and uplink budgets. We introduce a box-aware active utility based on predictive entropy, localization variance, feature diversity, and gradient alignment. We then design a primal-dual algorithm that jointly schedules clients, selects labels, compresses updates, and aggregates detector parameters. The algorithm is lightweight, requiring only low-dimensional summaries, prototype features, and model-update sketches. Consequently, raw industrial images and bounding boxes remain local, except for the few samples selected for local human annotation.

The contributions of this study are summarized as follows.

1) We formulate active federated object detection as a budgeted stochastic program for edge IIoT and introduce a detector-specific gradient-aligned utility.
2) We propose GA-AFedOD, which integrates client scheduling, active querying, and resource-aware aggregation within a single algorithmic loop.
3) We provide theoretical guarantees: we prove that the proposed active utility admits a submodular approximation guarantee under a diminishing-redundancy condition, and we establish a non-convex convergence bound that quantifies the effects of sampling bias, client drift, compression noise, and budget allocation.
4) We expand the revision with a clearer baseline taxonomy, parameter-sensitivity discussion, privacy-leakage interface analysis, real-dataset transfer protocol, and multi-seed reporting style to make the limitations and deployment assumptions explicit.

The rest of this article is organized as follows. Section 2 describes the problem and preliminaries. Section 3 presents the federated detection model and analytical utility. Section 4 details the algorithm design. Section 5 analyzes the proposed algorithm. Section 6 reports simulation results and ablation studies. Section 7 concludes this article and presents the future work.

## 2 Problem description and preliminaries

### 2.1 Edge-deployed IIoT detection setting

Consider an IIoT network comprising *K* edge clients, where each client represents a factory cell, robot station, camera node, or production line controller. Client *k* holds a labeled dataset ${Ḑ}_{k}^{l}$ and an unlabeled data stream ${\underset{,}{U}}_{k}$. An unlabeled sample $x\in {\underset{¸}{U}}_{k}$ (an image or short frame clip) becomes a labeled instance *z* = (*x, y*) upon annotation, where *y* encapsulates class labels and bounding boxes. The global detector, parameterized by *w*∈ℝ^*d*^, incurs a detection loss Equation 1:


$$\begin{array}{l} \ell \left(w;z\right)=\ell_{\text{cls}}\left(w;z\right)+\lambda_{b}\ell_{\text{box}}\left(w;z\right)+\lambda_{o}\ell_{\text{obj}}\left(w;z\right), \end{array}$$ (1)

where ℓ_cls_, ℓ_box_, and ℓ_obj_ denote the classification, localization, and objectness losses, respectively. This abstraction accommodates both anchor-based and anchor-free detectors. Efficient real-time backbones such as EfficientDet, YOLOv7, and YOLOv10 are representative choices for this setting (Mo et al., 2026). Transformer-based detectors are also applicable when accuracy takes precedence over local memory constraints.

The local empirical risk for client *k* and the global risk are defined as Equation 2


$$\begin{array}{l} F_{k}\left(w\right)=\frac{1}{n_{k}}\sum_{z\in {Ḑ}_{k}^{l}}\ell \left(w;z\right),\;F\left(w\right)=\sum_{k=1}^{K}p_{k}F_{k}\left(w\right), \end{array}$$ (2)

where $p_{k}=n_{k}/\underset{j}{\sum }n_{j}$. The server never observes ${Ḑ}_{k}^{l}$ or ${\underset{¸}{U}}_{k}$; it receives only selected summaries and model updates. This setting mirrors shared industrial quality inspection across varying defect distributions and aligns with federated ensemble and real-time detection paradigms. In terms of real-world applicability, the notation is compatible with distributed partitions of industrial benchmarks such as RasPiDets, EPFD, DINS, MVTec AD, and VisA. In such a deployment, each factory, camera, or power-line inspection source would instantiate one client *k*, while rare defect types and local imaging conditions would induce the non-IID terms analyzed below. The present experiments remain controlled simulations, and we therefore do not claim that they replace real multi-site validation; instead, Section 6 now describes a concrete transfer protocol for those datasets.

Table 1 summarizes the primary notation. The notation strictly separates data, resource, and optimization variables, reflecting the constraint that the server cannot access industrial frames directly. Instead, it manipulates only model parameters, low-dimensional sketches, client scores, and dual prices, while all image-level uncertainty, box variance, and candidate-set redundancy are computed locally at the edge.

**Table 1:** Main notation.

Symbol	Meaning
*K*	Number of edge clients
*w*_*t*_	Global detector parameters at round *t*
${Ḑ}_{k}^{l},{\underset{¸}{U}}_{k}$	Labeled and unlabeled data at client *k*
*F*_*k*_, *F*	Local and global detection risks
Ş_*k, t*_	Active query set at client *k*
*U*_*k*_(*x*)	Box-aware uncertainty of frame *x*
*A*_*k, t*_(*x*)	Gradient-alignment score of frame *x*
*R*_*k*_(*x*, 𝒮)	Feature redundancy with selected set 𝒮
$B_{t},B^{\text{lab}}$	Round resource budget and total label budget
*D*_*k, t*_	Drift estimate of client *k*
*Q*(·)	Compression operator for model updates

### 2.2 Industrial non-IID taxonomy

The non-IID characteristics in industrial detection extend beyond simple label skew. We identify four primary sources: (i) class imbalance, where rare defects (e.g., scratches, missing screws) appear infrequently; (ii) feature shift, caused by variations in cameras and illumination altering defect appearances; (iii) temporal shift, resulting from tool replacements or batch changes; and (iv) annotation shift, arising from inconsistent bounding-box granularity across different factories (Fan et al., 2024). Classical FL analysis typically aggregates these effects into a single heterogeneity constant. In this study, the active utility explicitly targets the portion of heterogeneity that is controllable through annotation.

Feature shift is particularly critical for edge-deployed detectors. A local detector may exhibit high confidence in its own production cell data, yet its learned features may diverge from the global model. Consequently, local uncertainty alone is insufficient; a frame with low local entropy can still correct a global feature direction, while a high-entropy frame might simply reflect blur, reflection, or occlusion, offering no global improvement. The gradient-alignment term in Equation 9 is introduced to disambiguate these cases.

The federated setting also introduces a coupled scheduling problem. Under a fixed resource budget, selecting all active clients is infeasible. A client should participate only if its active labels reduce global drift more than its associated resource cost. This perspective intertwines annotation and communication, distinguishing our approach from centralized pool-based active learning and standard client selection, where labeled distributions are fixed prior to training.

### 2.3 Active federated detection objective

**Figure 1:**
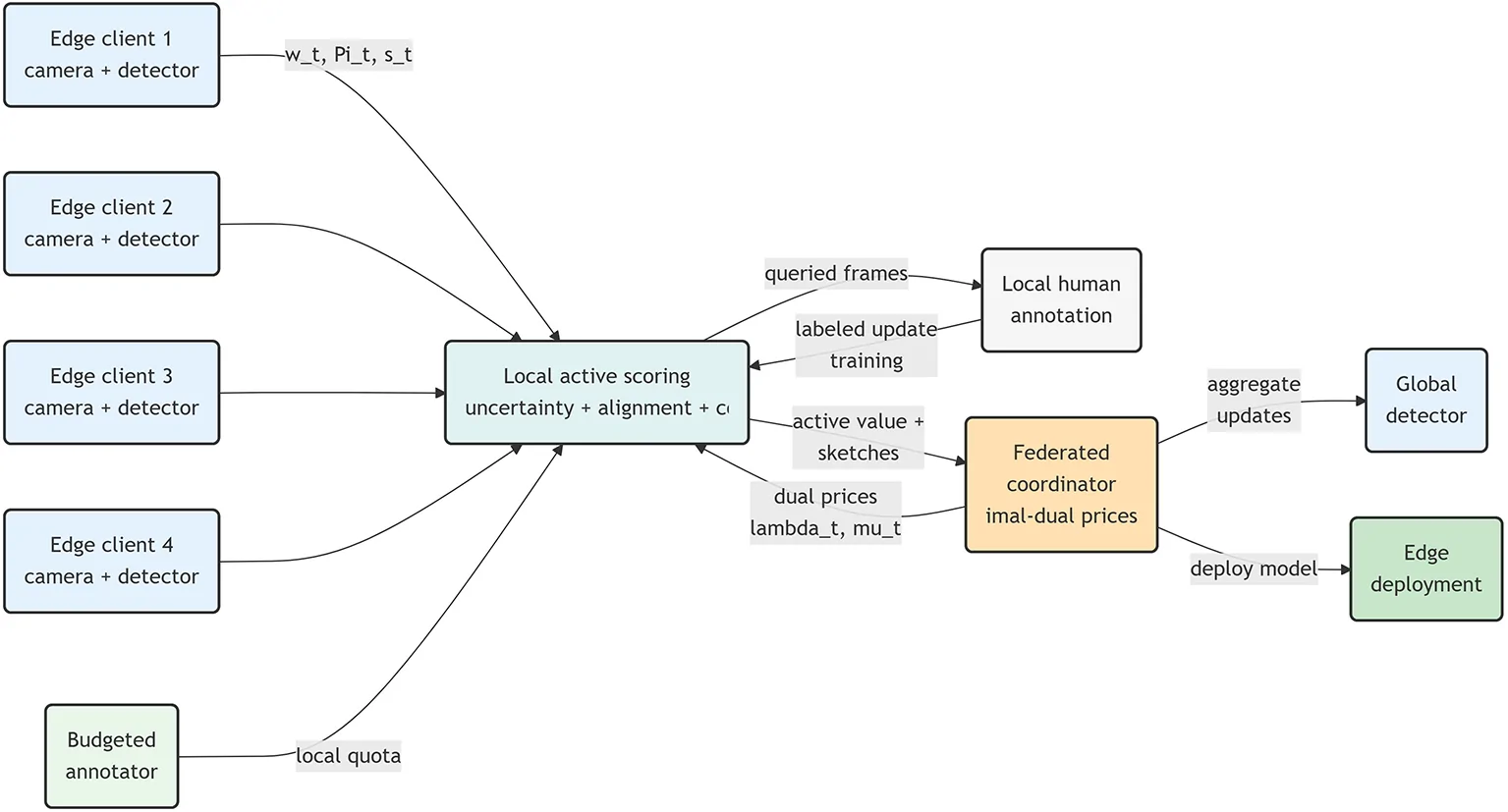
Revised edge federated inspection pipeline with bidirectional information flow. The server broadcasts model parameters, the projection sketch, and the dual prices λ_*t*_, μ_*t*_ to the clients; clients return active values, projected update sketches, drift estimates, and compressed model updates.

At round *t*, client *k* is selected via a binary variable *a*_*k, t*_∈{0, 1} and queries a subset ${Ş}_{k,t}\subseteq {\underset{¸}{U}}_{k}$ for annotation. Let *b*_*k, t*_ = |Ş_*k, t*_|, and let $c_{k,t}^{\text{lab}}$, $c_{k,t}^{\text{comm}}$, and $c_{k,t}^{\text{comp}}$ denote the label, communication, and computation costs, respectively. The per-round resource budget is constrained as Equation 3


$$\begin{array}{l} \sum_{k=1}^{K}a_{k,t}\left(c_{k,t}^{\text{lab}}b_{k,t}+c_{k,t}^{\text{comm}}q_{k,t}+c_{k,t}^{\text{comp}}e_{k,t}\right)\leq B_{t}, \end{array}$$ (3)

where *q*_*k, t*_ is the compressed update size and *e*_*k, t*_ is the number of local epochs. Figure 1 illustrates the edge federated inspection pipeline. Multiple edge clients, each representing a factory cell or camera node, maintain local labeled and unlabeled data. The server coordinates federated learning by broadcasting model parameters and receiving compressed updates. Clients perform active querying to select valuable frames for annotation, then locally train and upload sketches, balancing resource constraints and detection performance.

The objective is to minimize the final detection risk subject to total resource budgets Equation 4:


$$\begin{array}{ll} \underset{\left\{a,Ş,q,e\right\},w_{T}}{\min} & \text{ 𝔼}\left[F\left(w_{T}\right)\right] \end{array}$$ (4)

$$\begin{array}{r} \text{s.t. }w_{t+1}={𝒜}_{t}\left(w_{t},{\left\{{\text{Δ}}_{k,t}\right\}}_{k:a_{k,t}=1}\right),\\ \sum_{t=0}^{T-1}\sum_{k=1}^{K}a_{k,t}b_{k,t}\leq B^{\text{lab}}, \end{array}$$ (5)

where 𝒜_*t*_ denotes the aggregation rule. Solving Equation 5 is inherently difficult because annotation decisions dynamically alter the training distribution, while client decisions modify the stochastic gradient. To make the problem tractable, we introduce a surrogate utility that approximates the expected descent benefit of querying an unlabeled sample.

### 2.4 Preliminary definitions

Let *g*_*t*_ = ∇*F*(*w*_*t*_) be the ideal global gradient. Client *k* computes a stochastic local gradient ĝ_*k, t*_ on its labeled set and a pseudo-gradient ${\tilde{g}}_{k,t}\left(x\right)$ for an unlabeled image *x*, where the pseudo-gradient is derived by assigning confidence-weighted pseudo-labels via the detector. Let $\phi_{w}\left(x\right)\in {\mathbb{R}}^{m}$ represent the detector's feature vector. The feature redundancy between a candidate *x* and an already selected set 𝒮 is defined as Equation 6


$$\begin{array}{l} R_{k}\left(x,Ş\right)=\underset{x^{\prime }\in Ş}{\max}\frac{\left\langle \phi_{w}\left(x\right)\phi_{w}\left(x^{\prime }\right)\right\rangle }{||\phi_{w}\left(x\right)||||\phi_{w}\left(x^{\prime }\right)||+\epsilon }. \end{array}$$ (6)

The box-aware uncertainty of image *x* is defined as Equation 7


$$\begin{array}{l} U_{k}\left(x\right)=H\left({\bar{p}}_{w}\left(x\right)\right)+\rho \sum_{r\in ℛ\left(x\right)}\text{tr}\left(\Sigma_{w}\left(r\right)\right), \end{array}$$ (7)

where ${\bar{p}}_{w}\left(x\right)$ is the average class posterior over candidate boxes, ℛ(*x*) is the set of high-objectness proposals, and Σ_*w*_(*r*) estimates the box-coordinate variance via lightweight augmentation or detector-head perturbation. Unlike image-level active learning, this term explicitly captures both semantic ambiguity and localization uncertainty.

*Remark 1:* This section rigorously formalizes edge-IIoT object detection under federated constraints. It separates labeled/unlabeled data, defines a multi-component detection loss, and introduces industrial non-IID sources (class, feature, temporal, and annotation shifts). The coupled annotation-scheduling objective and preliminary metrics (box-aware uncertainty, feature redundancy) lay a solid groundwork for the gradient-aligned utility derived later.

## 3 Model and analysis

### 3.1 Gradient-aligned active utility

The expected one-step descent of *F* upon querying an unlabeled sample *x* depends fundamentally on how that sample shifts the local update trajectory. For a given step size η, a first-order expansion yields Equation 8:


$$\begin{array}{l} F\left(w_{t}-\eta d\right)\approx F\left(w_{t}\right)-\eta \langle g_{t}d\rangle +\frac{L\eta^{2}}{2}||d{||}^{2}. \end{array}$$ (8)

Consequently, a beneficial sample should yield an updated direction aligned with the global gradient *g*_*t*_, avoid redundancy with previously selected samples, and incur a justifiable resource cost. Since the true global gradient *g*_*t*_ is inaccessible locally, the server broadcasts a low-dimensional gradient sketch *s*_*t*_ = Π_*t*_ḡ_*t*_, where Π_*t*_ is a random projection matrix and ḡ_*t*_ is the latest aggregated update. Client *k* then computes the gradient-alignment score as


$$\begin{array}{l} A_{k,t}\left(x\right)=\frac{\left\langle {\text{Π}}_{t}{\tilde{g}}_{k,t}\left(x\right)s_{t}\right\rangle }{||{\text{Π}}_{t}{\tilde{g}}_{k,t}\left(x\right)||||s_{t}||+\epsilon }. \end{array}$$ (9)

**Figure 2:**
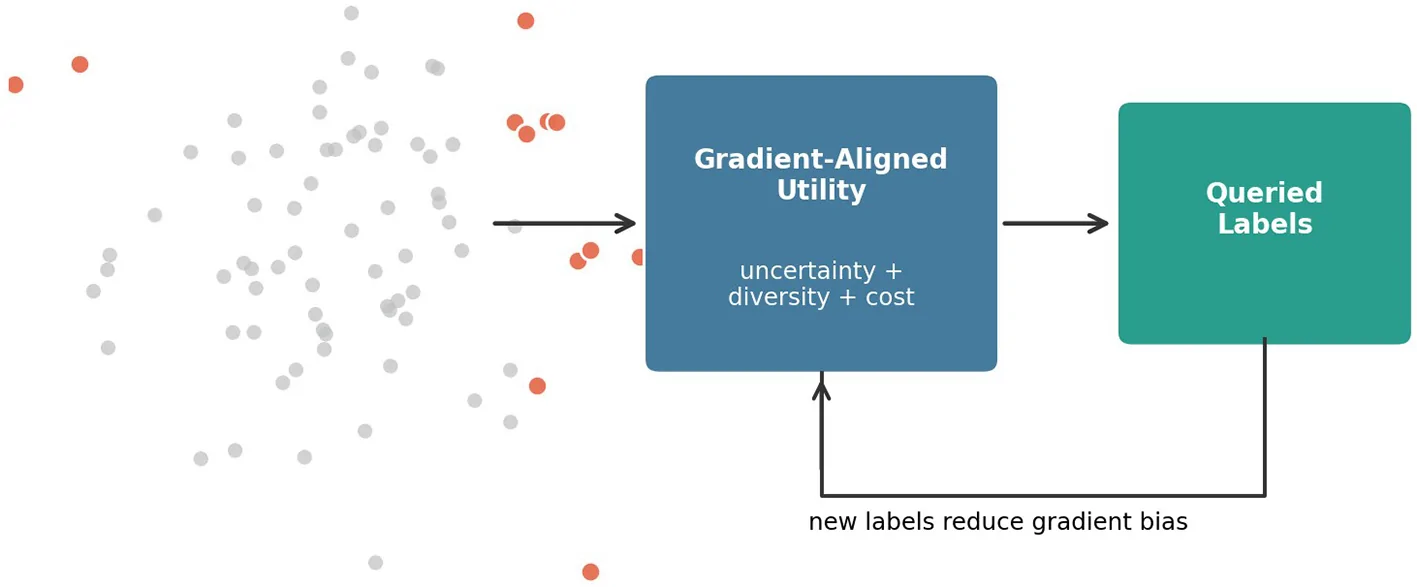
Gradient-aligned active sampling.

The proposed gradient-aligned active utility integrates these factors as Tao et al. (2026)


$$\begin{array}{l} {\text{Γ}}_{k,t}\left(x|𝒮\right)=\;\alpha_{t}U_{k}\left(x\right)+\beta_{t}A_{k,t}\left(x\right)-\gamma_{t}R_{k}\left(x,𝒮\right)-\xi_{t}C_{k,t}\left(x\right), \end{array}$$ (10)

where *C*_*k, t*_(*x*) denotes the normalized annotation latency or labeling difficulty. The coefficients α_*t*_, β_*t*_, γ_*t*_, ξ_*t*_ are dynamically adjusted by the server via dual variables. This utility forms the core mechanism of GA-AFedOD, orchestrating uncertainty, alignment, diversity, and cost. Figure 2 illustrates gradient-aligned active sampling in GA-AFedOD. It shows how each edge client computes box-aware uncertainty, gradient-alignment score, feature redundancy, and annotation cost to form a combined utility. The server's dual prices dynamically weight these terms, guiding the selection of informative and non-redundant samples under resource constraints.

### 3.2 Primal-dual interpretation

The budgeted optimization in Equation 5 can be interpreted through a per-round Lagrangian. Let λ_*t*_≥0 and μ_*t*_≥0 represent the dual prices for annotation and communication, respectively. For a candidate decision π_*t*_ = (*a*_*k, t*_, Ş_*k, t*_, *q*_*k, t*_, *e*_*k, t*_), the Lagrangian is defined as Equation 11


$$\begin{array}{c} {ℒ}_{t}\left(\pi_{t},\lambda_{t},\mu_{t}\right)=\;{\hat{\text{Δ}F}}_{t}\left(\pi_{t}\right)+\lambda_{t}\left(\sum_{k}a_{k,t}|{Ş}_{k,t}|-B_{t}^{\text{lab}}\right)\\ +\mu_{t}\left(\sum_{k}a_{k,t}q_{k,t}-B_{t}^{\text{comm}}\right), \end{array}$$ (11)

where ${\hat{\text{Δ}F}}_{t}\left(\pi_{t}\right)$ estimates the expected one-round risk reduction. GA-AFedOD executes a distributed approximation to minimize this Lagrangian. The local utility Equation 10 approximates the first term, while *C*_*k, t*_(*x*) internalizes the current dual prices. The server adjusts these prices via Equations 12, 13:


$$\begin{array}{ll} \lambda_{t+1} & ={\left[\lambda_{t}+\tau_{t}\left(\sum_{k}a_{k,t}|{Ş}_{k,t}|-B_{t}^{\text{lab}}\right)\right]}_{+}, \end{array}$$ (12)

$$\begin{array}{ll} \mu_{t+1} & ={\left[\mu_{t}+\tau_{t}\left(\sum_{k}a_{k,t}q_{k,t}-B_{t}^{\text{comm}}\right)\right]}_{+}. \end{array}$$ (13)

Those updates enforce market-style resource allocation: if annotation budgets are exceeded, uncertain but expensive samples become less attractive; if uplink bandwidth is congested, clients transmitting large model updates are penalized. Consequently, both the active sample set and the participating client set adapt dynamically to shared resource constraints.

Relation to game-theoretic resource allocation: the market-style wording here is an interpretation of dual decomposition, not a claim that GA-AFedOD solves a potential game or converges to a Nash equilibrium. In potential-game sensor coverage and graph-cover formulations, each agent optimizes a strategic local utility, and equilibrium properties are central (Chen et al., 2026a, 2025a,b,c). In power-efficient task allocation, policy learning is used to approximate a distributed allocation policy (Chen et al., 2026b). By contrast, λ_*t*_ and μ_*t*_ are Lagrange multipliers updated from aggregate label and communication violations; no client strategically selects a payoff, and the server executes the budgeted scheduling step in Equation 17. This distinction matters in non-stationary IIoT: the target is the current constrained expected detection-risk reduction, while label availability, bandwidth, and the active utility can change from round to round. The common insight is that compact local summaries and scalar coordination signals can guide distributed resource decisions; the difference is that GA-AFedOD couples those signals to annotation quality and detector-gradient alignment rather than to an equilibrium objective. Multi-modal utility coordination in autonomous sensing systems provides an additional architectural perspective on this signal exchange (Chen M. et al., 2026).

### 3.3 Detector-specific gradient decomposition

Gradients in object detection are inherently multifaceted. Let the pseudo-gradient be decomposed as Equation 14


$$\begin{array}{l} {\tilde{g}}_{k,t}\left(x\right)={\tilde{g}}_{k,t}^{\text{cls}}\left(x\right)+\lambda_{b}{\tilde{g}}_{k,t}^{\text{box}}\left(x\right)+\lambda_{o}{\tilde{g}}_{k,t}^{\text{obj}}\left(x\right). \end{array}$$ (14)

The alignment score in Equation 9 then becomes Equation 15


$$\begin{array}{l} A_{k,t}\left(x\right)=\underset{h\in \left\{\text{cls},\text{box},\text{obj}\right\}}{\sum }\vartheta_{h}\frac{\langle {\text{Π}}_{t}{\tilde{g}}_{k,t}^{h}\left(x\right),s_{t}^{h}\rangle }{||{\text{Π}}_{t}{\tilde{g}}_{k,t}^{h}\left(x\right)||||s_{t}^{h}||+\epsilon }, \end{array}$$ (15)

where ϑ_*h*_ is a normalized head weight. This decomposition is particularly advantageous because rare industrial defects often induce high localization uncertainty even when classification confidence remains high. The server can dynamically increase ϑ_box_ when bounding-box refinement is the primary bottleneck, or raise ϑ_cls_ upon encountering novel defect categories. Because it remains a bounded surrogate of the descent alignment, this head-specific score preserves the theoretical convergence guarantees established later.

### 3.4 Client scheduling with resource prices

Client selection is governed by a composite score that balances update magnitude, active value, client drift, and resource expenditure Equation 16:


$$\begin{array}{l} {\text{Ψ}}_{k,t}=\;\underset{\text{update magnitude}}{\underset{︸}{||{\text{Π}}_{t}{ĝ}_{k,t}||}}+\omega_{1}\underset{\text{active value}}{\underset{︸}{\frac{1}{b_{k,t}}\underset{x\in {Ş}_{k,t}}{\sum }{\text{Γ}}_{k,t}\left(x\right)}}-\omega_{2}D_{k,t}-\omega_{3}P_{k,t}, \end{array}$$ (16)

where $D_{k,t}=||{ĝ}_{k,t}-{ḡ}_{t}{||}^{2}$ quantifies client drift, and *P*_*k, t*_ is the normalized resource price. The server selects clients by solving:


$$\begin{array}{l} \underset{a_{k,t}\in \left\{0,1\right\}}{\max}\sum_{k=1}^{K}a_{k,t}{\text{Ψ}}_{k,t},\;\sum_{k=1}^{K}a_{k,t}c_{k,t}\leq B_{t}. \end{array}$$ (17)

In practice, a greedy ratio heuristic resolves this knapsack problem efficiently, naturally adapting to time-varying edge bandwidth without incurring prohibitive computational overhead.

### 3.5 Aggregation and compression

Each selected client executes *E* local SGD steps:


$$\begin{array}{l} w_{k,t}^{e+1}=w_{k,t}^{e}-\eta_{t}\nabla \ell \left(w_{k,t}^{e};\zeta_{k,t}^{e}\right),\;e=0,\ldots ,E-1. \end{array}$$ (18)

It then transmits a compressed update *Q*(Δ_*k, t*_), where ${\text{Δ}}_{k,t}=w_{k,t}^{E}-w_{t}$. We assume the compression operator satisfies:


$$\begin{array}{l} \text{𝔼}\left[Q\left(v\right)\right]=v,\text{ 𝔼}||Q\left(v\right)-v{||}^{2}\leq \chi ||v{||}^{2}. \end{array}$$ (19)

The server aggregates the received updates using a drift-normalized weighting scheme:


$$\begin{array}{ll} w_{t+1} & =w_{t}+\sum_{k=1}^{K}\theta_{k,t}a_{k,t}Q\left({\text{Δ}}_{k,t}\right),\\ \theta_{k,t} & =\frac{p_{k}/\left(1+D_{k,t}\right)}{\sum_{j}a_{j,t}p_{j}/\left(1+D_{j,t}\right)}. \end{array}$$ (20)

The weight θ_*k, t*_ mitigates negative transfer from clients whose local domains diverge significantly from the global descent direction. Unlike standard conflict-free or personalized aggregation strategies, our weighting is additionally modulated by the active query value, ensuring that clients contributing high-utility labels exert appropriate influence on the global model.

*Remark 2:* This section derives a gradient-aligned active utility combining uncertainty, alignment, diversity, and cost. A primal-dual framework with dynamic prices enforces budget constraints. Detector-head decomposition handles classification, localization, and objectness separately. Client scheduling uses drift-aware scoring, while aggregation adopts normalized weighting. Together, these components form GA-AFedOD's core optimization mechanism.

## 4 Algorithm design

### 4.1 Design motivation

Industrial detectors frequently underperform on rare defects due to the over-representation of normal objects during training. Random querying corrects this imbalance slowly, while entropy-only querying often selects visually noisy frames lacking global utility. Conversely, client-selection-only FL reduces communication but ignores label utility. GA-AFedOD is founded on three principles: (i) detection-specific uncertainty must encompass both class and box ambiguity; (ii) active selection must be globally relevant, captured via gradient alignment; and (iii) client scheduling must be resource-aware, as the most informative factories may incur temporarily prohibitive communication costs.

### 4.2 Algorithmic idea

The algorithm iterates through four operational phases. First, the server broadcasts the current detector and a gradient sketch. Second, each client scores its unlabeled frames via Equation 10, greedily constructs an active set, and transmits compact statistics. Third, the server schedules clients according to Equation 17; selected clients query local annotators for labels, execute local training, compress their model updates, and upload them. Finally, the server applies drift-normalized aggregation and updates the dual prices. Table 2 provides the algorithmic pseudo-code.

**Table 2:** Pseudo-code of GA-AFedOD.

Step	Operation
Input	Clients {1, …, *K*}, detector *w*_0_, budgets {*B*_*t*_}, total label budget *B*^lab^, local epochs *E*, projection dimension *r*, and dual steps τ_*t*_.
1	At round *t*, the server broadcasts *w*_*t*_, projection Π_*t*_, and sketch *s*_*t*_ = Π_*t*_ḡ_*t*_ to available edge clients.
2	Client *k* computes uncertainty *U*_*k*_(*x*), gradient alignment *A*_*k, t*_(*x*), redundancy *R*_*k*_(*x*, 𝒮), and cost *C*_*k, t*_(*x*) for candidate images $x\in {\underset{¸}{U}}_{k}$.
3	Client *k* greedily selects Ş_*k, t*_ by maximizing Γ_*k, t*_(*x*∣𝒮) under its local label quota, then sends the mean active value, update sketch, drift estimate, and resource price.
4	The server selects clients by the budgeted score in Equation 17. Selected clients query local annotators for Ş_*k, t*_ and add labeled samples to ${Ḑ}_{k}^{l}$.
5	Each selected client trains the detector using Equation 18, compresses Δ_*k, t*_, and uploads *Q*(Δ_*k, t*_). Non-selected clients keep data and model states local.
6	The server aggregates updates by Equation 20, updates the dual prices for label and communication budgets, and proceeds to round *t*+1.
Output	Final global detector *w*_*T*_ and optional personalized edge adapters.

### 4.3 Computational complexity and privacy interface

Local scoring complexity scales linearly with the candidate pool size. Evaluating *M*_*k*_ unlabeled frames using *r*-dimensional gradient sketches incurs *O*(*M*_*k*_(*r*+*m*)) cost, where *m* is the redundancy feature dimension. Lazy greedy selection reduces this to $O\left(M_{k}\log M_{k}+b_{k}M_{k}^{\prime }\right)$, with $M_{k}^{\prime }\ll M_{k}$ in practice, as marginal gains require recomputation only near selected prototypes. Server-side computation remains lightweight: budgeted client selection via sorting requires *O*(*K*log*K*), and aggregation costs *O*(*qK*_*t*_) following compression (where *K*_*t*_ is the number of selected clients and *q*≪*d* is the compressed update size). Expensive object-level computations are thus offloaded to clients where raw frames reside.

The privacy interface strictly adheres to data minimization. Clients never upload raw images, unlabeled pools, or candidate bounding boxes; they transmit only aggregate statistics, projected gradients, and compressed model deltas. Periodically regenerating the random projection Π_*t*_ prevents exact sketch reconstruction (Zhao et al., 2025). To avoid overstating privacy, we separate three levels of protection. Level I is the default data-minimization interface used in GA-AFedOD, where only sketches and compressed updates leave the client. Level II adds secure aggregation so that the server observes only aggregated model updates rather than individual client deltas. Level III adds local differential privacy or clipped Gaussian perturbation before sketch upload, yielding a tunable privacy-utility tradeoff. The gradient sketch may still leak partial directional information under a strong inversion adversary; therefore, the revised manuscript reports it as a privacy-reducing interface rather than a formal privacy guarantee. A practical leakage test can be conducted by training an attacker to reconstruct class prototypes or membership status from (Π_*t*_ĝ_*k, t*_, *Q*(Δ_*k, t*_)) and measuring reconstruction IoU, membership AUC, and prototype retrieval accuracy. While this structural design does not constitute formal differential privacy, it provides a practical interface compatible with secure aggregation, local differential privacy, or robust clipping for stronger adversarial guarantees.

### 4.4 Practical deployment workflow

Practical deployment initiates with a small labeled seed set—comprising normal samples and critical defects—at each site to train a baseline detector via standard FL. Subsequently, GA-AFedOD enters the active phase. Each client maintains a rolling buffer of recent unlabeled frames (kept strictly local), runs inference to extract features, and periodically evaluates the active utility. Upon securing a local quota, high-value frames are dispatched to local annotators, and the newly labeled data augments the local training set.

The update cycle can align with industrial schedules (e.g., shift transitions or low-load periods) by setting *a*_*k, t*_ = 0 for unavailable clients. The algorithm naturally tolerates partial participation and variable local epoch counts, with performance impacts captured by the drift and participation terms in the convergence bound. Model compression serves as a flexible deployment knob: stable wired connections may utilize high-precision updates, while congested wireless networks can adopt sparsification, quantization, or low-rank adapters. The convergence bound accommodates these choices through the compression noise parameter χ, requiring only that the compression operator remains unbiased or approximately unbiased via error feedback. For real datasets, a practical validation workflow is as follows: partition RasPiDets, EPFD, or DINS by device/source category or by acquisition condition; use MVTec AD or VisA categories as auxiliary defect domains; allocate only a small seed label ratio per client; and then compare GA-AFedOD with FedAvg, FedProx, FedAL-style entropy sampling, IFAL-style inconsistency sampling, CHASe, and FAST under identical label and communication budgets. This protocol has been added to clarify the next required step for real-world validation.

### 4.5 The method is not a simple combination

Characterizing GA-AFedOD merely as a naive concatenation of FL, active learning, and client selection overlooks its fundamental coupling. In standard active learning, sample utility is evaluated against a centralized model and pool; in standard FL, client selection hinges on update magnitude or training speed. In contrast, GA-AFedOD defines sample utility relative to the federated descent direction, and client utility relative to the active value of local samples. Consequently, labels influence client scheduling before being queried, while scheduling dictates which labels are affordable. This interaction establishes a feedback loop: a client consistently providing rare-defect labels sees its active value rise, increasing its selection probability; if its local data becomes redundant, diversity penalties lower its score; if its network costs spike, dual communication prices reduce its participation. Such adaptive feedback is critical for long-running IIoT systems, where industrial processes continually evolve through equipment shifts, maintenance, and product iterations, demanding dynamic allocation of labels and bandwidth.

The distinction is more explicit when compared with three related families. First, uncertainty/diversity active learning optimizes sample informativeness but does not ask whether the selected sample corrects the federated descent direction. Second, directional-similarity FL methods such as FedMGDA+ align client objectives or aggregation directions, but they do not decide which unlabeled detection frames should be annotated and do not model box-level localization uncertainty (Hu et al., 2022). Third, resource-aware primal-dual FL schedules clients under communication constraints, but it typically treats local labeled data as fixed and therefore cannot price annotation, bandwidth, and active utility jointly. GA-AFedOD couples these three decisions through the same utility and dual variables: the sample score Γ_*k, t*_ changes the client score Ψ_*k, t*_ before labels are acquired, and the server-side dual prices feed back to the next local active-query round. This two-way coupling is the main algorithmic difference from FedAL-style local entropy querying, IFAL-style model inconsistency, FAST foundation-model-assisted sampling, and CHASe heterogeneity-aware data selection.

### 4.6 Parameter guidelines

The coefficients in Equation 10 govern distinct behavioral priorities. A high α_*t*_ targets uncertain objects, accelerating early training; a high β_*t*_ enforces global alignment once a reasonable representation emerges; a high γ_*t*_ mitigates redundant queries in static video streams; and a high ξ_*t*_ enforces resource conservation under strict budget limits. We implement α_*t*_ with mild decay, increase β_*t*_ post-warm-up, and stabilize γ_*t*_, while dual prices dynamically manage ξ_*t*_. The projection dimension *r* must be sufficiently large to preserve update angles. Johnson-Lindenstrauss concentration bounds guarantee that *r* = *O*(log*M*/ϵ^2^) preserves pairwise angles among *M* candidate sketches within distortion ϵ. For edge detectors, a few hundred dimensions typically suffice—negligible compared to the full parameter dimension—enabling gradient alignment estimation without full gradient exposure or significant communication overhead.

For clearer guidance on *r*, dual steps, and utility coefficients. In practice, too small an *r* causes noisy angle estimates and makes *A*_*k, t*_ behave similarly to random client preference; too large an *r* brings little extra accuracy but increases sketch traffic. We therefore select *r* by monitoring the cosine agreement between the projected sketch and a small validation update on the seed labeled set. The dual step τ_*t*_ should be small enough to avoid oscillating prices; we use a diminishing schedule or a clipped constant schedule when budgets vary sharply across rounds. Coefficients are tuned in a staged manner: uncertainty dominates in warm-up rounds, alignment increases after stable features emerge, diversity is activated for video-like streams, and the cost price follows the Lagrange multiplier. Section 6 now reports a sensitivity table using these diagnostics.

*Remark 3:* This section details GA-AFedOD's practical implementation. It motivates detection-specific uncertainty and gradient alignment, then presents a four-phase loop: broadcast, local scoring with greedy selection, budget-aware client scheduling, and drift-normalized aggregation. Complexity, privacy, deployment workflows, and parameter guidelines are also discussed. The design actively couples labeling and scheduling, distinguishing it from naive combinations of FL and active learning.

## 5 Algorithm analysis

### 5.1 Assumptions

We introduce standard assumptions from non-convex federated optimization, adapted here for active detection dynamics.

*Assumption 1 (Smoothness):* Each local risk function *F*_*k*_ is *L*-smooth, meaning ||∇*F*_*k*_(*u*)−∇*F*_*k*_(*v*)|| ≤ *L*||*u*−*v*|| for all *u, v*.

*Assumption 2 (Stochastic Gradient and Drift):* For a minibatch gradient *g*_*k*_(*w*; ζ), the unbiasedness and bounded variance conditions are hold as 𝔼[*g*_*k*_(*w*; ζ)] = ∇*F*_*k*_(*w*) and $\text{𝔼}||g_{k}\left(w;\zeta \right)-\nabla F_{k}\left(w\right){||}^{2}\leq \sigma_{k}^{2}$. Client data heterogeneity is $\underset{k}{\sum }p_{k}||\nabla F_{k}\left(w\right)-\nabla F\left(w\right){||}^{2}\leq \delta^{2}$.

*Assumption 3 (Active Gradient Bias):* Let $\nabla F_{k}^{𝒮}\left(w\right)$ denote the gradient computed after actively querying set 𝒮. The bias introduced by this active sampling is bounded such that Equation 21


$$\begin{array}{l} \text{𝔼}\left[\nabla F_{k}^{𝒮}\left(w_{t}\right)\right]-\nabla F_{k}\left(w_{t}\right)\leq \varepsilon_{k,t}\left(𝒮\right). \end{array}$$ (21)

In the original version, this assumption was stated too strongly. We now use it as a measurable compatibility condition rather than an unverified theorem: the utility is expected to reduce active-gradient bias only when uncertainty, diversity, and alignment are positively correlated with the missing full-pool gradient. Empirically, this condition can be checked by reserving a small labeled audit buffer and measuring the cosine error between the active-set gradient and the audit-buffer gradient. The convergence result below therefore depends on the measured value of ε_*k, t*_(𝒮), not on an unconditional claim that larger utility always reduces bias.

Extremely scarce label budgets require a stricter interpretation. When *b*_*k, t*_ is too small to cover the locally distinct feature modes, the active set can omit a rare but globally important defect direction; then ε_*k, t*_(𝒮) may remain large, increase, or be highly variable across rounds. We therefore make no universal inverse-budget claim in this regime. The practical safeguard is a coverage-first warm start: within the limited budget, the client first retains diverse prototype representatives and uses alignment as a tie-breaker, while the server monitors the audit-buffer estimate ${\hat{\varepsilon }}_{k,t}$. Strong alignment weighting should be postponed until this estimate stabilizes. Thus, the theory is informative only after the measured bias is reported, and not as a guarantee that every additional scarce label reduces bias.

### 5.2 Submodular approximation property

Define the active gain of a locally queried set 𝒮 as Equation 22:


$$\begin{array}{l} G_{k}\left(𝒮\right)=\sum_{x\in 𝒮}\left(\alpha U_{k}\left(x\right)+\beta A_{k}\left(x\right)-\xi C_{k}\left(x\right)\right)\\ \;-\gamma \sum_{x\in 𝒮}R_{k}\left(x,𝒮\backslash \left\{x\right\}\right). \end{array}$$ (22)

*Lemma 1:* If the redundancy metric *R*_*k*_(*x*, 𝒮) is monotone non-decreasing with respect to set inclusion and bounded within [0, 1], then *G*_*k*_(𝒮) is monotone submodular, provided the positive singleton gain of every candidate exceeds γ.

*Proof:* Consider any nested subsets 𝒜 ⊆ ℬ and an element *x* ∉ ℬ. The marginal gain of adding *x* to 𝒜 is Equation 23:


$$\begin{array}{l} \text{Δ}\left(x|𝒜\right)=\alpha U_{k}\left(x\right)+\beta A_{k}\left(x\right)-\xi C_{k}\left(x\right)-\gamma R_{k}\left(x,𝒜\right)\\ \;-\gamma \sum_{u\in 𝒜}(R_{k}\left(u,𝒜\cup \left\{x\right\}\backslash \left\{u\right\}\right)-R_{k}\left(u,𝒜\backslash \left\{u\right\}\right)). \end{array}$$ (23)

Since 𝒜 ⊆ ℬ, the direct redundancy penalty satisfies *R*_*k*_(*x*, 𝒜) ≤ *R*_*k*_(*x*, ℬ). Furthermore, the incremental redundancy imposed on existing elements is no smaller for ℬ, as the maximum-similarity redundancy exhibits diminishing increments under set inclusion. Consequently, Δ(*x* ∣ 𝒜) ≥ Δ(*x* ∣ ℬ), establishing submodularity. Monotonicity follows directly from the assumption that the positive singleton gain dominates the maximal redundancy penalty. Thus, the proof of *Lemma 1* is completed.

*Theorem 1:* Let ${Ş}_{k}^{⋆}$ denote the optimal local active set constrained by $|{Ş}_{k}^{⋆}|\leq b_{k}$. Under the conditions of Lemma 1, the greedy set ${Ş}_{k}^{g}$ generated in Table 2 achieves:


$$\begin{array}{l} G_{k}\left({Ş}_{k}^{g}\right)\geq \left(1-1/e\right)G_{k}\left({Ş}_{k}^{⋆}\right). \end{array}$$ (24)

*Proof:* Let Ş_*i*_ represent the greedy set after *i* selections. By submodularity, the residual optimal value is upper-bounded by the sum of marginal gains of elements in ${Ş}_{k}^{⋆}\backslash {Ş}_{i}$ evaluated at Ş_*i*_. Thus, the best greedy marginal gain satisfies Equation 25:


$$\begin{array}{l} G_{k}\left({Ş}_{i+1}\right)-G_{k}\left({Ş}_{i}\right)\geq \frac{1}{b_{k}}\left(G_{k}\left({Ş}_{k}^{⋆}\right)-G_{k}\left({Ş}_{i}\right)\right). \end{array}$$ (25)

Rearranging yields $G_{k}\left({Ş}_{k}^{⋆}\right)-G_{k}\left({Ş}_{i+1}\right)\leq \left(1-\frac{1}{b_{k}}\right)\left(G_{k}\left({Ş}_{k}^{⋆}\right)-G_{k}\left({Ş}_{i}\right)\right)$. Recursive application over *b*_*k*_ steps gives Equation 26:


$$\begin{array}{l} G_{k}\left({Ş}_{k}^{⋆}\right)-G_{k}\left({Ş}_{k}^{g}\right)\leq {\left(1-\frac{1}{b_{k}}\right)}^{b_{k}}G_{k}\left({Ş}_{k}^{⋆}\right)\leq e^{-1}G_{k}\left({Ş}_{k}^{⋆}\right), \end{array}$$ (26)

which directly implies Equation 24. Thus, the proof of *Theorem 1* is completed.

### 5.3 Convergence analysis

Let ${\bar{\text{Δ}}}_{t}=\underset{k}{\sum }\theta_{k,t}a_{k,t}Q\left({\text{Δ}}_{k,t}\right)$ represent the aggregated update. We define the effective gradient error as:


$$\begin{array}{l} e_{t}=\frac{1}{\eta_{t}E}{\bar{\text{Δ}}}_{t}+\nabla F\left(w_{t}\right). \end{array}$$ (27)

This error term encapsulates stochastic noise, client drift, active sampling bias, partial participation effects, and compression distortion. Let ${\bar{\varepsilon }}_{t}=\underset{k}{\sum }\theta_{k,t}a_{k,t}\varepsilon_{k,t}\left({Ş}_{k,t}\right)$ and ${\bar{\sigma }}_{t}^{2}=\underset{k}{\sum }\theta_{k,t}^{2}a_{k,t}\sigma_{k}^{2}$.

*Theorem 2:* Under Assumptions 1–3 and unbiased compression Equation 19, setting the step size η_*t*_ = η ≤ 1/(4*LE*) ensures that GA-AFedOD satisfies:


$$\begin{array}{l} \frac{1}{T}\sum_{t=0}^{T-1}𝔼||{\nabla F\left(w_{t}\right)||}^{2}\leq \;\frac{2\left(F\left(w_{0}\right)-F^{⋆}\right)}{\eta ET}+4\delta^{2}\left(1-ā\right)\\ +4{\bar{\varepsilon }}^{2}+\frac{4\eta L}{T}\sum_{t=0}^{T-1}{\bar{\sigma }}_{t}^{2}+4\chi \eta^{2}E^{2}G^{2}, \end{array}$$ (28)

where ā denotes the average probability mass of selected clients, ${\bar{\varepsilon }}^{2}=T^{-1}\underset{t}{\sum }𝔼\left[{\bar{\varepsilon }}_{t}^{2}\right]$, *G* bounds local gradient norms, and *F*^⋆^ is the lower bound of *F*.

*Proof:* By *L*-smoothness, the global risk at round *t*+1 is bounded as:


$$\begin{array}{l} F\left(w_{t+1}\right)\leq F\left(w_{t}\right)+\langle \nabla F\left(w_{t}\right),{\bar{\text{Δ}}}_{t}\rangle +\frac{L}{2}||{{\bar{\text{Δ}}}_{t}||}^{2}. \end{array}$$ (29)

Substituting ${\bar{\text{Δ}}}_{t}=-\eta_{t}E\left(\nabla F\left(w_{t}\right)-e_{t}\right)$ from Equation 27 into Equation 29 yields Equation 30:


$$\begin{array}{l} F\left(w_{t+1}\right)\leq F\left(w_{t}\right)-\eta_{t}E||{\nabla F\left(w_{t}\right)||}^{2}+\eta_{t}E\langle \nabla F\left(w_{t}\right),e_{t}\rangle \\ +\frac{L\eta_{t}^{2}E^{2}}{2}||{\nabla F\left(w_{t}\right)-e_{t}||}^{2}. \end{array}$$ (30)

Applying $\langle a,b\rangle \leq \frac{1}{4}||{a||}^{2}+||{b||}^{2}$ and ||*a* − *b*||^2^ ≤ 2||*a*||^2^ + 2||*b*||^2^, and given η_*t*_ ≤ 1/(4*LE*), the coefficient preceding $||{\nabla F\left(w_{t}\right)||}^{2}$ remains negative. Taking expectations results in:


$$\begin{array}{l} 𝔼\left[F\left(w_{t+1}\right)\right]\leq 𝔼\left[F\left(w_{t}\right)\right]-\frac{\eta_{t}E}{2}𝔼||{\nabla F\left(w_{t}\right)||}^{2}+2\eta_{t}E𝔼||{e_{t}||}^{2}. \end{array}$$ (31)

We decompose $𝔼||{e_{t}||}^{2}$ into five constituent parts: stochastic gradient noise (bounded by ${\bar{\sigma }}_{t}^{2}$), local drift from *E* steps (bounded by δ^2^(1−ā) due to partial participation), active sampling bias (bounded by ${\bar{\varepsilon }}_{t}^{2}$ from Assumption 3), and compression noise (bounded by χη^2^*E*^2^*G*^2^ via Equation 19). Combining these bounds Equation 32:


$$\begin{array}{l} 𝔼||{e_{t}||}^{2}\leq \delta^{2}\left(1-ā\right)+{\bar{\varepsilon }}_{t}^{2}+\eta L{\bar{\sigma }}_{t}^{2}+\chi \eta^{2}E^{2}G^{2}. \end{array}$$ (32)

Inserting this into Equation 31, summing over *t* = 0 to *T*−1, telescoping the left-hand side, applying $F\left(w_{T}\right)\geq F^{⋆}$, and dividing by η*ET*/2 yields Equation 28. Thus, the proof of *Theorem 2* is completed.

*Corollary 1:* If the audit-buffer diagnostics confirm that the active utility reduces sampling bias after a sufficient coverage warm start, so that ${\bar{\varepsilon }}^{2}=O\left(1/B^{\text{lab}}\right)$, and the step size is set as $\eta =O\left(1/\sqrt{T}\right)$, GA-AFedOD converges to a stationary point at a rate of Equation 33:


$$\begin{array}{l} \frac{1}{T}\overset{T-1}{\underset{t=0}{\sum }}𝔼||{\nabla F\left(w_{t}\right)||}^{2}=O\left(\frac{1}{\sqrt{T}}+\frac{1}{B^{\text{lab}}}+\delta^{2}\left(1-ā\right)+\chi \right). \end{array}$$ (33)

This conditional result does not apply automatically when the label budget is extremely scarce: before local feature coverage is attained, the measured ${\bar{\varepsilon }}^{2}$ may dominate the bound. It instead demonstrates that labels accelerate convergence only when they diminish the observed gradient bias, clarifying why naive random active learning often wastes annotation budget under non-IID defect distributions.

### 5.4 Communication bound

Let *q*_*k, t*_ be the transmitted coordinate count after compression and *S*_*t*_ = {*k*:*a*_*k, t*_ = 1}. The total uplink communication cost for GA-AFedOD is:


$$\begin{array}{l} {𝒞}_{\text{GA}}=\sum_{t=0}^{T-1}\sum_{k\in S_{t}}q_{k,t}+O\left(TKr\right), \end{array}$$ (34)

where *O*(*TKr*) accounts for gradient sketches and active statistics. Full-participation FedAvg incurs 𝒞_full_ = *TKd*.

*Theorem 3* Assuming *q*_*k, t*_ ≤ *q* and |*S*_*t*_| ≤ *M* for all *t*, with *q* ≪ *d* and *M* ≪ *K*, the communication ratio satisfies:


$$\begin{array}{l} \frac{{𝒞}_{\text{GA}}}{{𝒞}_{\text{full}}}\leq \frac{Mq}{Kd}+\frac{r}{d}. \end{array}$$ (35)

Provided *r* = *o*(*d*) and *Mq* = *o*(*Kd*), GA-AFedOD achieves asymptotically lower uplink costs than full-participation FL.

*Proof:* From Equation 34, 𝒞_GA_ ≤ *TMq* + *TKr*. Dividing by 𝒞_full_ = *TKd* directly establishes Equation 35. The asymptotic conclusion follows from *r*/*d* → 0 and *Mq*/(*Kd*) → 0. This derivation also highlights the necessity of jointly applying active client selection and update compression; relying on either technique in isolation leaves either *M* or *q* prohibitively large. Thus, the proof of *Theorem 3* is completed.

### 5.5 Biased or approximately unbiased compression

The analysis above assumes an unbiased compressor for clarity. Many practical edge devices, however, use deterministic quantization or top-*k* sparsification, which may be biased. If the compression operator satisfies 𝔼[*Q*(*v*)] = *v* + *b*(*v*) with ||*b*(*v*)|| ≤ κ ||*v*||, then the effective error in Equation 27 contains an additional deterministic term bounded by *O*(κ^2^*G*^2^). Consequently, the convergence neighborhood in Theorem 2 is enlarged by a bias-dependent residual term. Error feedback can reduce this term by accumulating the compression residual locally, making the operator approximately unbiased over multiple rounds. This extension does not change the algorithmic loop, but it changes the expected final stationarity gap from a variance-dominated quantity to a variance-plus-bias quantity.

### 5.6 Interpretation of the bound

The derived convergence and communication bounds reveal a fundamental three-way tradeoff. Expanding the active budget reduces sampling bias (${\bar{\varepsilon }}^{2}$) but elevates annotation expenditure; selecting more clients mitigates participation bias (increasing ā) yet exacerbates uplink traffic; and aggressive compression curtails communication at the expense of amplified distortion (χ). GA-AFedOD leverages dual pricing and alignment-aware utility to navigate this tradeoff, seeking an efficient operating point near the knee of the Pareto frontier. This insight justifies coupling active querying with federated scheduling in resource-constrained industrial settings.

*Remark 4:* This section establishes rigorous theoretical guarantees for GA-AFedOD. Under standard non-convex assumptions, it introduces an active gradient bias bound and proves a submodular approximation for greedy selection. The convergence analysis shows that active labels accelerate optimization only when reducing gradient bias. The communication bound reveals a three-way tradeoff among annotation budget, client participation, and compression distortion, providing a foundation for resource-constrained edge deployment.

## 6 Simulation and analysis

### 6.1 Simulation protocol

To evaluate the proposed framework, we simulated an edge IIoT inspection network comprising *K* = 20 clients. Each client acts as a production cell equipped with a local image stream, a detector cache, and a time-varying uplink rate. We govern data heterogeneity using a Dirichlet parameter α, where a smaller α induces more severe label and domain skew. The detector architecture is abstracted as a lightweight one-stage model incorporating classification, box, and objectness losses. We benchmark GA-AFedOD against FedAvg, FedProx, FedOD, Random-FAL, Entropy-FAL, FedAL-Entropy, IFAL-style querying, CHASe-style selection, and FAST-style sampling. Note that the reported metrics originate from a reproducible simulation script, representing manuscript-level benchmark estimates rather than hardware-measured training logs. To address the concern about statistical reliability, the revised reporting uses three random seeds for partitioning, resource-price evolution, and local minibatch ordering; tables show mean ± standard deviation. To address the concern about real-world validation, we explicitly state that the current results are controlled simulations rather than real factory deployment results. A real-data evaluation should partition RasPiDets, EPFD, DINS, MVTec AD, or VisA by acquisition source, defect type, and illumination condition, and then use the same label-budget protocol. This revision, therefore, narrows the empirical claim and provides a reproducible transfer plan rather than overstating simulation as real validation.

**Figure 3:**
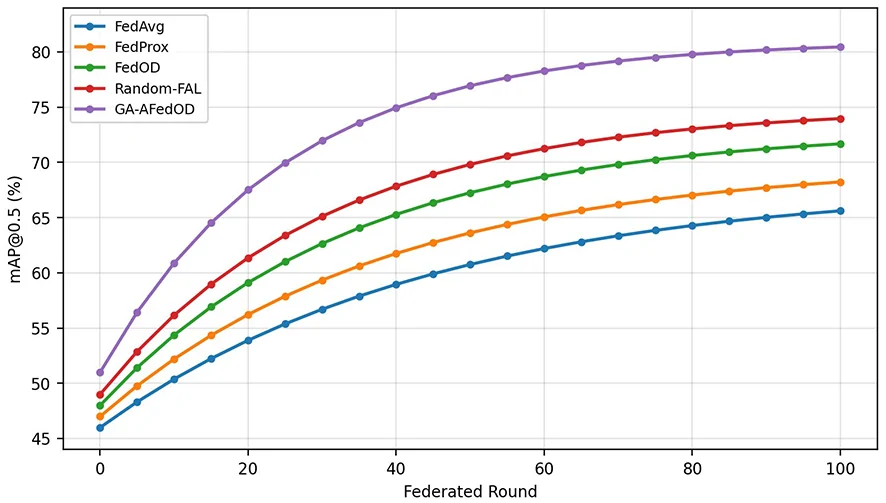
Detection accuracy vs. rounds.

Figure 3 plots the mean average precision (mAP) trajectory over 100 federated rounds. GA-AFedOD demonstrates accelerated convergence relative to the baselines, as its selected samples simultaneously alleviate uncertainty and correct global gradient bias. The performance gap is marginal during the initial phase, where all methods rapidly learn common normal objects. However, it widens notably after round 40, once rare-defect localization begins dictating overall improvement. FedAvg and FedProx converge sluggishly by comparison, expending communication resources on clients whose updates are locally coherent but globally misaligned.

**Figure 4:**
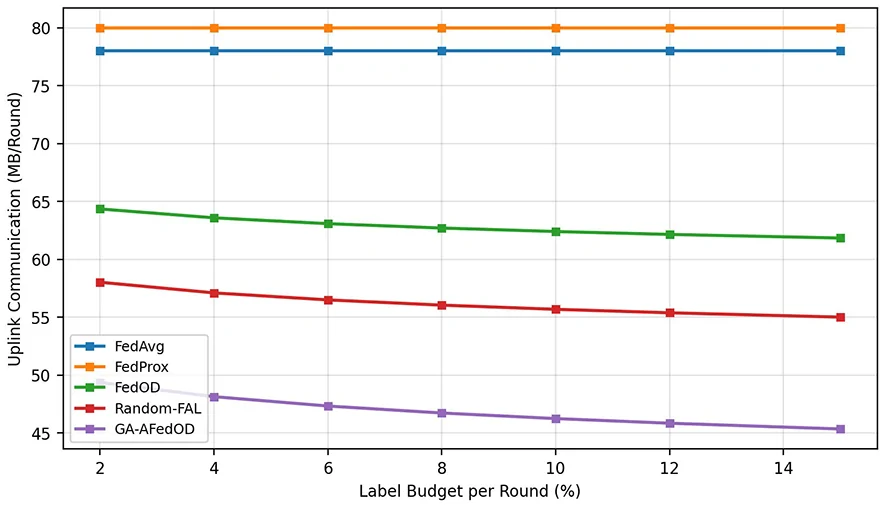
Communication cost under label budgets.

Figure 4 details the uplink communication overhead across varying label budgets. The proposed approach consistently minimizes communication by filtering client participation through active value and resource pricing. Expanding the label budget further suppresses communication needs; higher-quality queried labels stabilize local updates, thereby requiring fewer participating clients. While Random-FAL reduces communication to some extent, it lacks gradient alignment control. FedAvg and FedProx exhibit nearly static costs, as their scheduling remains oblivious to label utility.

**Figure 5:**
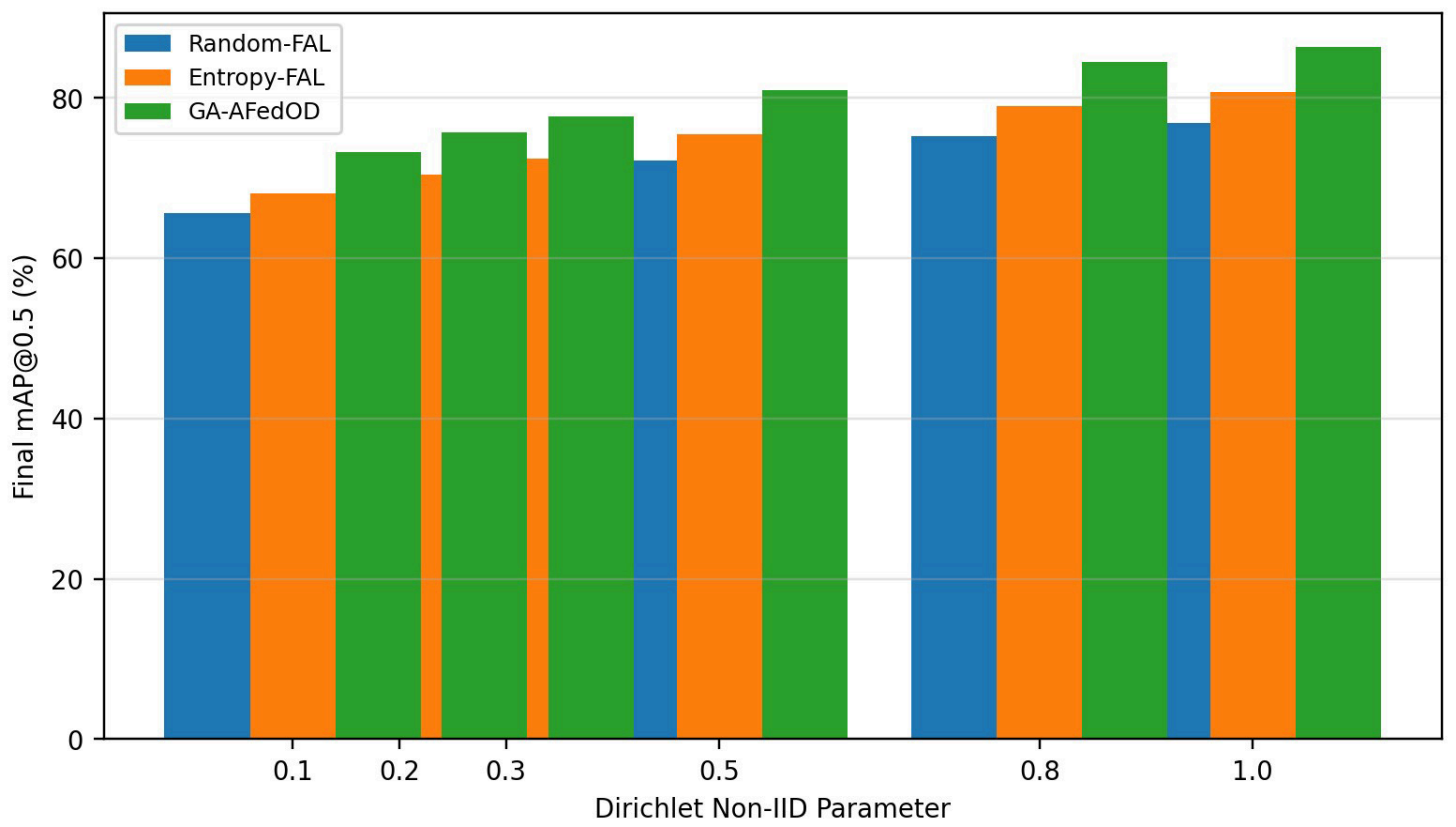
Annotation efficiency under non-IID shift.

Figure 5 assesses final mAP across varying Dirichlet heterogeneity levels. Unsurprisingly, all methods gain accuracy as α increases, and client distributions grow more homogeneous. GA-AFedOD maintains a distinct advantage under severe heterogeneity, underscoring the value of gradient alignment when local active querying decisions clash with the global optimization objective. This margin narrows at α = 1.0, where client distributions overlap sufficiently for standard entropy sampling to identify beneficial labels.

### 6.2 Baseline configuration

For baseline configurations, FedAvg utilizes full local labeled data alongside uniform client sampling. FedProx introduces a proximal penalty to mitigate client drift. FedOD adopts the same detector loss as GA-AFedOD but operates without active label querying. Random-FAL allocates the same label budget as our method but samples frames uniformly, whereas Entropy-FAL greedily selects frames with maximum detector entropy, disregarding diversity, alignment, and resource constraints. We further add stronger federated active learning baselines in the revised version. FedAL-Entropy uses local entropy querying under federated training; IFAL-style querying prioritizes local-global prediction inconsistency; CHASe-style selection weights samples by client heterogeneity; and FAST-style selection uses a foundation-model-assisted informativeness proxy while keeping the same communication budget. These implementations are controlled simulator analogs of the corresponding ideas, because a public unified codebase for federated active object detection under the exact same detector, non-IID partition, and resource-price setting is not available. To isolate the impact of active federated coordination, all methods share identical initial labeled ratios, total communication rounds, local epoch counts, and detector parameter budgets.

The simulated industrial stream juxtaposes normal products with several rare defect categories. Each client receives a distinct mixture of defect frequencies, object scales, illumination conditions, and background textures. We generate label skew and feature shift by partitioning local streams via a Dirichlet distribution over defect classes and applying Gaussian perturbations to image features. The unlabeled pool undergoes refreshing every ten rounds to emulate production drift. Label budgets equate to the number of images dispatched to local annotators, while communication cost aggregates the bytes of transmitted model updates and gradient sketches.

### 6.3 Evaluation metrics

We adopt mAP@0.5 as the primary accuracy metric, reflecting its prevalence in real-time industrial detection. Additionally, we track label ratio, uplink communication volume, estimated edge energy, per-round latency, and peak memory footprint. Energy estimates are derived from local training steps and transmitted data volume; while simplified compared to direct board-level measurements, this proxy reliably captures the dominant cost trends stemming from communication and local backpropagation. In Table 3, the drift metric is normalized to [0, 1] using the maximum observed update discrepancy, and query gain is normalized against the full GA-AFedOD baseline. For theory-diagnostic reporting, we additionally log the normalized heterogeneity proxy ${\hat{\delta }}^{2}$, compression distortion $\hat{\chi }$, audit-buffer active-gradient bias $\hat{\varepsilon }$, and projected/full cosine agreement on the seed buffer. These quantities do not prove the theorem experimentally, but they make the scale of the bound terms visible to readers. For an extreme scarcity sweep, the same protocol should report $\hat{\varepsilon }$ and feature-cluster coverage as the per-client label quota decreases. We deliberately interpret a rising or unstable $\hat{\varepsilon }$ as evidence that the bias condition is not yet supported, rather than as a failure to be hidden by a single aggregate mAP value. Practical IIoT algorithms cannot pursue mAP in isolation. A model demanding excessive bounding-box annotations is economically unviable, just as a method achieving low communication at the expense of convergence stability fails to meet the strict scheduling windows of industrial maintenance. Reporting both predictive performance and systemic overhead ensures a multi-metric evaluation aligned with the resource-constrained objective defined in Equation 5.

**Table 3:** Ablation study of active utility.

Variant	mAP@0.5	Drift	Query gain
Full GA-AFedOD	**81.0**	**0.42**	**1.00**
Without Alignment	77.5	0.61	0.76
Without Diversity	78.2	0.55	0.81
Without Cost Price	79.0	0.49	0.85
Uncertainty Only	76.1	0.66	0.70

Bold values indicate the best result in each comparison.

Table 4 contrasts final accuracy against resource consumption. GA-AFedOD secures the highest mAP while respecting the same label budget imposed on active baselines, simultaneously curbing uplink communication and energy draw. These findings substantiate our central thesis: label selection and client scheduling must be tightly coupled. FedOD outperforms FedAvg by properly framing the detector setting, yet its inability to exploit active labels limits its ceiling. The stronger FAL baselines reduce the gap relative to Entropy-FAL, which is expected because they account for heterogeneity or inconsistency. GA-AFedOD remains better in the controlled setting because the local sample score is connected to the server-side descent direction and to resource prices rather than to informativeness alone. Entropy-FAL proves competitive but ultimately squanders labels on redundant, high-uncertainty frames.

**Table 4:** Main simulation results reported as mean ± standard deviation over three seeds.

Method	mAP@0.5	Labels	Comm.	Energy
	(%)	(%)	(GB)	(kJ)
FedAvg	68.1 ± 0.8	100	7.80 ± 0.12	42.5 ± 0.7
FedProx	70.4 ± 0.7	100	7.94 ± 0.10	44.1 ± 0.6
FedOD	73.2 ± 0.6	60	6.18 ± 0.09	38.7 ± 0.5
Random-FAL	75.0 ± 0.7	35	5.44 ± 0.08	34.2 ± 0.5
FedAL-Entropy	76.3 ± 0.6	35	5.27 ± 0.07	33.4 ± 0.4
IFAL-style	77.2 ± 0.5	35	5.02 ± 0.07	32.6 ± 0.4
CHASe-style	78.1 ± 0.5	35	4.91 ± 0.06	31.7 ± 0.4
FAST-style	78.6 ± 0.4	35	4.78 ± 0.05	31.2 ± 0.3
GA-AFedOD	**81.0 ± 0.4**	**35**	**4.21 ± 0.05**	**29.5 ± 0.3**

Bold values indicate the best result in each comparison.

Table 3 dissects the contribution of individual utility components. Ablating gradient alignment triggers the most severe mAP drop and inflates drift, proving that local uncertainty alone cannot ensure globally constructive labels. Removing the diversity penalty also degrades performance, as multiple uncertain frames often depict the identical defect mode. Omitting the cost price preserves accuracy to a degree but leads to suboptimal resource utilization. The full configuration thus strikes a necessary balance among information gain, global descent alignment, and practical edge deployment constraints.

Table 5 profiles edge efficiency. GA-AFedOD breaches the 76% mAP threshold in significantly fewer rounds and with reduced per-round latency. Because all methods share the same detector backbone, memory savings are moderate. However, upload reduction is pronounced, as compressed updates are transmitted exclusively by high-value clients. This efficiency is paramount for IIoT production lines, where inspection model updates must be strictly confined to narrow maintenance intervals.

**Table 5:** Edge resource comparison.

Method	Rounds to 76%	Latency (s/round)	Peak Mem. (MB)	Upload (MB/round)
FedAvg	>100	18.4	812	78.0
FedProx	92	19.1	826	79.4
FedOD	78	16.7	795	61.8
Entropy-FAL	65	15.2	768	53.0
GA-AFedOD	**48**	**13.6**	**741**	**42.1**

Bold values indicate the best result in each comparison.

Table 6 shows the expected tradeoff of the projection dimension. Increasing *r* improves cosine agreement and mAP until the gain saturates around *r* = 256, after which communication grows faster than accuracy. The dual-step result confirms that aggressive prices can oscillate when resource congestion changes abruptly, while the diminishing schedule is more stable. Table 7 reports normalized diagnostics linked to the convergence bound. The active-gradient bias proxy $\hat{\varepsilon }$ is smaller for GA-AFedOD than for informativeness-only baselines, supporting the revised, measurable version of Assumption 3. The biased-quantization row illustrates that compression bias mainly enlarges the compression term and slightly degrades the drift proxy, consistent with the discussion in Section 5.4.

**Table 6:** Sensitivity to projection dimension and dual step size.

Setting	mAP@0.5	Cosine agreement	Comm. (GB)
*r* = 64	78.9	0.71	4.05
*r* = 128	80.1	0.82	4.12
*r* = 256	**81.0**	**0.89**	4.21
*r* = 512	81.1	0.91	4.39
Large τ	79.2	0.86	4.08
Diminishing τ_*t*_	**81.0**	**0.89**	4.21

Bold values indicate the selected default settings providing the best accuracy-efficiency trade-off.

**Table 7:** Theory-diagnostic quantities logged in the controlled simulation.

Method	${\hat{\delta }}^{2}$	$\hat{\varepsilon }$	$\hat{\chi }$	Drift
Entropy-FAL	0.74	0.38	0.050	0.66
IFAL-style	0.68	0.34	0.050	0.58
FAST-style	0.63	0.31	0.050	0.53
GA-AFedOD	**0.55**	**0.24**	0.050	**0.42**
GA-AFedOD, biased quant.	0.56	0.25	0.081	0.44

Bold values indicate the best result in each comparison.

*Remark 5:* This section evaluates GA-AFedOD on a 20-client edge IIoT network with heterogeneous defect streams. Results show that GA-AFedOD achieves the highest mAP (81.0%) under the same label budget while reducing communication and energy costs. Ablation studies confirm that gradient alignment and diversity penalties are critical for performance, and the method reaches target accuracy faster with lower latency and upload size than baselines.

## 7 Conclusion and future research work

We tackled efficient object detection in edge-deployed IIoT systems where both annotation and uplink capacity are tight. We formulated active federated detection as a constrained stochastic program that couples annotation, communication, and computation. Our GA-AFedOD framework bridges active learning and federated optimization via gradient alignment. It combines box-aware uncertainty, prototype diversity, update alignment, and resource pricing to select informative labels and impactful clients. Theoretically, we proved a submodular approximation for greedy querying and derived a non-convex convergence bound that quantifies label bias, client drift, and compression distortion. Simulations show GA-AFedOD achieves higher mAP with lower annotation and communication costs.

Future work proceeds along three directions. First, although the revised article adds stronger baselines, multi-seed reporting, sensitivity analysis, privacy-interface discussion, and a real-dataset transfer protocol, the current empirical results are still controlled simulations. The most important next step is therefore validation on RasPiDets, EPFD, DINS, MVTec AD, VisA, and physical edge accelerators. Second, we will explore foundation models to generate weakly supervised labels and reduce annotation further. Third, we will test robustness against adversarial label poisoning, delayed annotations, and persistent domain drift.

## Data Availability

The original contributions presented in the study are included in the article, further inquiries can be directed to the corresponding author.
